# Air Pollution Exposure and Abnormal Glucose Tolerance during Pregnancy: The Project Viva Cohort

**DOI:** 10.1289/ehp.1307065

**Published:** 2014-02-07

**Authors:** Abby F. Fleisch, Diane R. Gold, Sheryl L. Rifas-Shiman, Petros Koutrakis, Joel D. Schwartz, Itai Kloog, Steven Melly, Brent A. Coull, Antonella Zanobetti, Matthew W. Gillman, Emily Oken

**Affiliations:** 1 Division of Endocrinology, Boston Children’s Hospital, Boston, Massachusetts, USA; 2 Channing Laboratory, Brigham and Women’s Hospital, Boston, Massachusetts, USA; 3 Department of Environmental Health, Harvard School of Public Health, Boston, Massachusetts, USA; 4 Obesity Prevention Program, Department of Population Medicine, Harvard Medical School and Harvard Pilgrim Health Care Institute, Boston, Massachusetts, USA; 5 Department of Geography and Environmental Development, Ben-Gurion University of the Negev, Beer Sheva, Israel; 6 Department of Biostatistics, and; 7 Department of Nutrition, Harvard School of Public Health, Boston, Massachusetts, USA

## Abstract

Background: Exposure to fine particulate matter (PM with diameter ≤ 2.5 μm; PM_2.5_) has been linked to type 2 diabetes mellitus, but associations with hyperglycemia in pregnancy have not been well studied.

Methods: We studied Boston, Massachusetts–area pregnant women without known diabetes. We identified impaired glucose tolerance (IGT) and gestational diabetes mellitus (GDM) during pregnancy from clinical glucose tolerance tests at median 28.1 weeks gestation. We used residential addresses to estimate second-trimester PM_2.5_ and black carbon exposure via a central monitoring site and spatiotemporal models. We estimated residential traffic density and roadway proximity as surrogates for exposure to traffic-related air pollution. We performed multinomial logistic regression analyses adjusted for sociodemographic covariates, and used multiple imputation to account for missing data.

Results: Of 2,093 women, 65 (3%) had IGT and 118 (6%) had GDM. Second-trimester spatiotemporal exposures ranged from 8.5 to 15.9 μg/m^3^ for PM_2.5_ and from 0.1 to 1.7 μg/m^3^ for black carbon. Traffic density was 0–30,860 vehicles/day × length of road (kilometers) within 100 m; 281 (13%) women lived ≤ 200 m from a major road. The prevalence of IGT was elevated in the highest (vs. lowest) quartile of exposure to spatiotemporal PM_2.5_ [odds ratio (OR) = 2.63; 95% CI: 1.15, 6.01] and traffic density (OR = 2.66; 95% CI: 1.24, 5.71). IGT also was positively associated with other exposure measures, although associations were not statistically significant. No pollutant exposures were positively associated with GDM.

Conclusions: Greater exposure to PM_2.5_ and other traffic-related pollutants during pregnancy was associated with IGT but not GDM. Air pollution may contribute to abnormal glycemia in pregnancy.

Citation: Fleisch AF, Gold DR, Rifas-Shiman SL, Koutrakis P, Schwartz JD, Kloog I, Melly S, Coull BA, Zanobetti A, Gillman MW, Oken E. 2014. Air pollution exposure and abnormal glucose tolerance during pregnancy: the Project Viva Cohort. Environ Health Perspect 122:378–383; http://dx.doi.org/10.1289/ehp.1307065

## Introduction

Air pollution, especially fine particulate matter (PM_2.5_), which is composed of particles with an aerodynamic diameter ≤ 2.5 μm, may promote insulin resistance [reviewed by Rajagopalan and Brook (2012)]. PM_2.5_ results from combustion and is a constituent of automobile exhaust and power plant emissions. It is composed of black (elemental) carbon, organic carbon, sulfates, nitrates, metals, dust, and biological material. Because of its small size, PM_2.5_ readily enters the bronchi and alveoli. PM_2.5_ has been associated with local and systemic inflammation and adverse cardiorespiratory outcomes. For example, PM_2.5_-exposed rodents developed pulmonary (Happo et al. 2012) and systemic (Wang et al. 2013) inflammation and impaired cardiovascular function (Wang et al. 2013). In population-based human studies, higher PM_2.5_ exposure was associated with increased systemic inflammation in pregnant women (Lee et al. 2011) and increased cardiorespiratory hospitalizations in nonpregnant adults (Dominici et al. 2006).

In high-fat-diet and normal-weight rodent models, PM_2.5_ exposure induced insulin resistance by promoting adipose inflammation and through potential disruption of insulin signal transduction (Sun et al. 2009; Xu et al. 2011). Several adult cohort studies have explored associations between long-term particulate matter exposure and self-reported type 2 diabetes mellitus (Andersen et al. 2012; Brook et al. 2008; Coogan et al. 2012; Krämer et al. 2010; Pearson et al. 2010; Puett et al. 2011), and all but one (Puett et al. 2011) reported positive associations between diabetes and particulate matter exposures.

Pregnancy is a particularly vulnerable time for the development of abnormal glycemia because insulin resistance increases as part of the normal physiological adaptation to ensure fuel transfer to the fetus. Up to 18% of pregnant women worldwide develop some degree of abnormal glucose tolerance by the end of the second trimester (Sacks et al. 2012). About one-third of these women meet current diagnostic criteria for gestational diabetes mellitus (GDM), and the remaining two-thirds have impaired glucose tolerance (IGT), a milder form of glucose intolerance (International Association of Diabetes Pregnancy Study Groups Consensus Panel et al. 2010), which, like GDM, is associated with adverse maternal (Retnakaran et al. 2008) and fetal (Hapo Study Cooperative Research Group et al. 2008) outcomes. In contrast with type 2 diabetes, which often develops over years and for which diagnosis is often delayed (Inzucchi 2012), insulin resistance in pregnancy develops during the second trimester, and screening routinely occurs at the end of this trimester (Butte 2000). Thus, when considering the effects of air pollution on glycemia, focusing on insulin resistance during pregnancy permits evaluation of acute, directly relevant exposure windows.

A cohort study in the Netherlands (van den Hooven et al. 2009) reported no association between traffic density and GDM, whereas a study of birth registry data in Sweden reported a monotonic dose–response association between nitrogen oxides (NO_x_) and GDM and positive associations with traffic density (Malmqvist et al. 2013). However, neither study measured PM_2.5_ or black carbon exposure, neither assessed more mild degrees of GDM, and the Swedish cohort did not include individual-level socioeconomic status covariate data.

The primary objective of the present analysis was to evaluate the association of second-trimester PM_2.5_ exposure, using two exposure assessment approaches, with glycemia in a large cohort of pregnant women residing in the greater Boston, Massachusetts, area. We hypothesized that PM_2.5_ exposure would be positively associated with IGT and GDM. Secondary objectives were to estimate associations with additional measures of exposure to traffic-related air pollution, including black carbon concentration, neighborhood traffic density, and home roadway proximity.

## Methods

*Study population and design*. From 1999 to 2002, we recruited Boston-area women at their first prenatal visit to Harvard Vanguard Medical Associates, a multi-specialty group practice with eight urban and suburban obstetric offices throughout eastern Massachusetts, to participate in the Project Viva cohort. Eligibility criteria for Project Viva included fluency in English, gestational age of ≤ 22 weeks at enrollment, and singleton pregnancy. A total of 2,128 women with a live birth were included in Project Viva; for the present analysis, we excluded 16 women with preexisting type 1 or type 2 diabetes mellitus and 19 women without any exposure measurements available. Of the remaining 2,093, the number included in each analysis varied from 1,584 to 2,092 based on the availability of exposure data (Table 1).

**Table 1:** Of 2,093 women eligible to be included in the analyses, sample sizes for each analysis varied based on the exposure method.

Exposure	Sample size	Inclusion criteria
Central-site PM_2.5_	1,943	Residential address within 40 km of the central monitoring site
Spatiotemporal PM_2.5_	1,584	Second trimester began after March 2000 (at which time satellite data became available)
Central-site black carbon	1,943	Residential address within 40 km of the central monitoring site
Spatiotemporal black carbon	2,069	Residential address within our spatiotemporal black carbon model area, which included eastern Massachusetts
Neighborhood traffic density	2,081	Residential address in Massachusetts mainland
Home roadway proximity	2,092	Residential address able to be geocoded

Participants provided their residential address at enrollment and updated it at the second study visit, timed to coincide with clinical glycemic screening (median, 28.1 weeks). We estimated exposures for all women who lived at an address in our catchment area for at least 75% of the second trimester. Geocoding and spatial analyses were done using ArcGIS version 10.1 and StreetMap^TM^ roads (ESRI, Redlands, CA, USA).

All participants provided written informed consent, and institutional review boards of the participating sites approved the study.

*Air pollution exposure assessments*. We measured daily PM_2.5_ and black carbon at a monitoring site located atop the Harvard University Countway Library in Boston, Massachusetts (Kang et al. 2010). We assigned these daily central site values to women living within 40 km of the monitor.

We also estimated PM_2.5_ and black carbon concentrations at each woman’s residential address using spatiotemporal models. Although estimated, these concentrations had the advantage of more closely matching a woman’s residential address than did central-site measurements. Also, spatiotemporal models allowed for spatial as well as temporal variability (i.e., two women pregnant at a similar time but living in different neighborhoods could have different exposures). For estimates of daily spatiotemporal PM_2.5_ exposure (Kloog et al. 2011), we used mixed-effects models with random slopes for day and nested regions to calibrate daily satellite aerosol optical depth (AOD) data (http://ladsweb.nascom.nasa.gov/index.html) at a resolution of a 10 × 10 km spatial grid (2000–2008) with all monitored PM_2.5_ measurements in New England. We then used a generalized additive mixed model with spatial smoothing and regional measured PM_2.5_, AOD values in neighboring cells, and land use variables to estimate PM_2.5_ for location-day pairs with missing AOD. The “out of sample” 10-fold cross-validation *R*^2^ for days with and without available AOD data was 0.83 and 0.81, respectively.

We estimated daily spatiotemporal black carbon exposure at each residential address using a validated spatiotemporal land use regression model (Gryparis et al. 2007) that included daily average black carbon estimates from 148 monitoring stations from January 1999 to August 2011. Predictors in the final model included address-specific land use, 2009 traffic density, daily meteorological factors, other seasonal characteristics, and their interactions. We also used data from the Boston central monitoring site to reflect daily variations in black carbon in the region. For each of the pollutants, we estimated second-trimester exposures by averaging daily concentrations from day 94 through day 187 after last menstrual period.

We estimated neighborhood traffic density [average daily traffic (vehicles/day) × length of road (kilometers) within 100 m] using the 2002 road inventory from the Massachusetts Executive Office of Transportation [as in Kloog et al. (2012); Zeka et al. (2008)]. Home roadway proximity (distance to census feature class code A1 or A2 roads) was calculated using U.S. and Canada detailed streets from Street Map^TM^ North America ArcGIS 10 Data and Maps (time period of content 2005; ArcGIS). For both variables we used residential address at study enrollment (median, 9.9 weeks gestation).

*Glycemic screening and classification of glucose tolerance status*. At the end of the second trimester of gestation (median, 28.1 weeks), participating women completed routine clinical screening for GDM (Herring et al. 2009). If serum glucose 1 hr after a nonfasting 50 g oral glucose challenge test (GCT) was ≥ 140 mg/dL, the participant was referred for a 3-hr fasting 100-g oral glucose tolerance test (OGTT). Normal OGTT results, per American Diabetes Association (ADA) criteria (American Diabetes Association 2008), were blood glucose ≤ 95 mg/dL at baseline, ≤ 180 mg/dL at 1 hr, ≤ 155 mg/dL at 2 hr, and ≤ 140 mg/dL at 3 hr. Given a combination of the GCT and OGTT results, we focused on two categories of glucose intolerance: *a*) We defined GDM as failing the GCT with ≥ 2 high values on the OGTT as per ADA criteria (American Diabetes Association 2008); and *b*) we defined IGT as failing the GCT (1-hr glucose result of ≥ 140 mg/dL) with one high value on the OGTT. Although there is not currently a uniformly recognized definition for IGT during pregnancy, this definition allowed for comparison with previously published work (Herring et al. 2009; Retnakaran et al. 2008; Saldana et al. 2006). The reference group [normal glucose tolerance (NGT)] comprised women with GCT results ≤ 140 mg/dL who did not have OGTT testing. We classified the remaining mothers who had GCT results ≥ 140 mg/dL but no high values on the OGTT as a separate outcome group because data are mixed regarding whether maternal and fetal outcomes for women with these laboratory results are different from women with NGT (Hillier et al. 2007; Retnakaran et al. 2008).

*Assessment of covariates*. Using a combination of interviews and questionnaires, we collected information on participants’ age, race/ethnicity, education, household income, history of GDM in a previous pregnancy, family history of diabetes mellitus, smoking habits, and date of the last menstrual period updated with ultrasound. We calculated prepregnancy body mass index (BMI; kilograms per meter squared) from self-reported height and weight. We calculated total gestational weight gain up to glycemic screening as the difference between the weight measured on the date of the glycemic screen and self-reported prepregnancy weight (Herring et al. 2009).

*Statistical analysis*. We used multinomial logistic regression analyses to evaluate associations of air pollution exposures with IGT and GDM. In each model, we estimated separate ORs for 4 possible outcomes: *a*) NGT, which we used as a common “reference” outcome; *b*) failed GCT normal OGTT; *c*) IGT; and *d*) GDM. We estimated separate odds ratios (ORs) for these outcomes because other studies have shown different predictors for IGT versus GDM (Hillier et al. 2007; Saldana et al. 2006).

We considered each of the exposures (central-site PM_2.5_, spatiotemporal PM_2.5_, central-site black carbon, spatiotemporal black carbon, traffic density, and distance to roadway) in separate models. We initially modeled PM_2.5_, black carbon, and traffic density exposures as categorical variables (in quartiles) to assess for potential nonlinearity of exposure–outcome relationships. We *a priori* dichotomized proximity to major roadway as > or ≤ 200 m for consistency with previous studies, using > 200 m as a reference group (Puett et al. 2011; van den Hooven et al. 2009). We first fit unadjusted models. Next we created a full multivariate model for each of the exposures that included as covariates maternal age (continuous), prepregnancy BMI (continuous), pregnancy weight gain through time of OGTT (continuous), race/ethnicity (white, black, Asian, Hispanic, other), education (with or without college degree), smoking habits (never, former, or during pregnancy), season of last menstrual period (4 seasons), prior GDM (yes, no, or nulliparous), family history of diabetes (yes or no), and household income (> $70,000 or ≤ $70,000). We then excluded household income and smoking habits because neither was a confounder of the relationship of any of the exposures with IGT or GDM (i.e., the estimate for the primary exposure changed by < 10%). Because categorical exposure–outcome relationships appeared linear, we also modeled PM_2.5_, black carbon, and traffic density exposures as continuous measures, and expressed associations per interquartile range (IQR) increase in exposure.

As is common in large epidemiologic analyses, many participants were missing data on one or more variables. We used chained equations to multiply impute missing values (White et al. 2011) [the MI procedure in SAS (SAS Institute Inc., Cary, NC, USA)]. We generated 50 imputed data sets, and all model results were generated by appropriately combining these results (Rubin 2004). To avoid incorrect imputations, we used all 2,128 cohort participants with live births and included all covariates as well as exposure and outcome variables in the imputation process (White et al. 2011). In the analytic data set, we included only participants with measured exposures (*n* = 2,093). In women missing outcome data (*n* = 43), we imputed outcomes in addition to covariates. Including imputed outcome data could add additional covariate information and would not be expected to bias regression results because exposure data were not imputed and outcomes were assumed to be missing at random (Little 1992).

We performed several sensitivity analyses. We individually included additional covariates for trends over time (based on the calendar date of the last menstrual period), prepregnancy BMI squared, and 1999 census tract median household income (based on residential address at enrollment) (U.S. Census Bureau 2000a) to the final model. We also limited the analysis to the subset of women with no history of prior GDM (*n* = 2,051) and the subset of women with a measured rather than imputed outcome (*n* = 2,050). Because spatiotemporal PM_2.5_ and traffic density were both significantly associated with IGT and were not highly collinear, we considered both exposures concomitantly in the final model. All analyses were conducted using SAS version 9.3 (SAS Institute Inc.).

## Results

Of the 2,093 women in the study population, 65 (3%) had IGT and 118 (6%) had GDM. Second-trimester mean ± SD (range) central-site PM_2.5_ was 10.9 ± 1.4 μg/m^3^ (8.3–17.2 μg/m^3^) and spatiotemporal PM_2.5_ was 11.9 ± 1.4 μg/m^3^ (8.5–15.9 μg/m^3^). Thus, we anticipated that PM_2.5_ annual averages in the study population were generally lower than the U.S. Environmental Protection Agency (2013) threshold for annual exposure, which was 15 μg/m^3^ at the time and was lowered to 12 μg/m^3^ in December 2012. Second-trimester mean ± SD (range) central-site black carbon was 0.9 ± 0.1 μg/m^3^ (0.6–1.1 μg/m^3^) and spatiotemporal black carbon was 0.7 ± 0.2 μg/m^3^ (0.1–1.7 μg/m^3^). Traffic density mean was 1,621 ± 2,234 (0–30,860) vehicles/day × km of road within 100 m; 272 (13%) of the women lived within 200 m of a major roadway. Central-site PM_2.5_ and black carbon were not correlated with traffic density or roadway proximity, and other exposures were moderately correlated (Spearman correlation coefficients 0.08–0.79) (see Supplemental Material, Table S1). Mean age at enrollment was 31.8 years, and mean prepregnancy BMI was 24.9 kg/m^2^ (Table 2). Only a small percentage of women had a family history of diabetes (8%) or prior GDM (2%). Imputation had little or no influence on the distribution of participant characteristics (see Supplemental Material, Table S2). Women recruited before March 2000, when satellite measurements became available, and who therefore were not included in analyses of spatiotemporal PM_2.5_, had lower central-site PM_2.5_ exposure and higher black carbon exposure but did not differ from other participants in terms of sociodemographic characteristics or the proportions of women with IGT or GDM (see Supplemental Material, Table S2). Of the covariates, only race/ethnicity varied by exposure status, with white women living more frequently at addresses with lower PM_2.5_, and black, Hispanic, and Asian women at addresses with higher PM_2.5_ exposure (Table 2).

**Table 2:** Characteristics of participants in Project Viva [mean ± SD or *n* (%)], overall^*a*^ and by spatiotemporal PM_2.5_ quartile (Q).^*b*^

Characteristic	Overall	PM_2.5_ Q1	PM_2.5_ Q2	PM_2.5_ Q3	PM_2.5_ Q4
Maternal age at enrollment (years)	31.8 ± 5.2	32.0 ± 4.9	31.7 ± 5.2	32.0 ± 5.0	31.9 ± 5.4
Prepregnancy BMI (kg/m^2^)	24.9 ± 5.6	24.8 ± 5.9	24.7 ± 5.1	24.8 ± 5.1	24.8 ± 5.6
Pregnancy weight gain to OGTT (kg)	10.2 ± 4.4	10.4 ± 4.3	10.0 ± 4.2	10.0 ± 4.4	10.5 ± 4.9
Central-site PM_2.5_ (μg/m^3^)	10.9 ± 1.4	9.9 ± 0.7	10.7 ± 0.7	11.5 ± 0.9	12.5 ± 1.3
Spatiotemporal PM_2.5_ (μg/m^3^)	11.9 ± 1.4	10.2 ± 0.5	11.3 ± 0.3	12.3 ± 0.3	13.8 ± 0.8
Central-site black carbon (μg/m^3^)	0.9 ± 0.1	0.8 ± 0.1	0.8 ± 0.1	0.8 ± 0.1	0.9 ± 0.1
Spatiotemporal black carbon (μg/m^3^)	0.7 ± 0.2	0.6 ± 0.2	0.7 ± 0.2	0.7 ± 0.2	0.8 ± 0.2
Traffic density [(vehicles/day) × km]	1,621 ± 2,234	1,368 ± 2,201	1,692 ± 2,256	1,722 ± 2,081	1,716 ± 2,237
Roadway proximity (≤ 200 m)	281 (13)	39 (10)	55 (14)	58 (15)	60 (15)
College graduate	1,354 (65)	260 (66)	258 (65)	264 (67)	257 (65)
Race/ethnicity
White	1,397 (67)	287 (73)	265 (67)	255 (64)	253 (64)
Black	345 (17)	51 (13)	68 (17)	58 (15)	69 (17)
Asian	118 (6)	15 (4)	22 (5)	31 (8)	21 (5)
Hispanic	153 (7)	23 (6)	28 (7)	34 (9)	40 (10)
Other	80 (4)	19 (5)	13 (3)	17 (4)	12 (3)
Family history of diabetes	165 (8)	32 (8)	25 (6)	33 (8)	38 (10)
Prior history of gestational diabetes
Yes	42 (2)	10 (3)	4 (1)	7 (2)	10 (3)
No	1,052 (50)	216 (55)	204 (51)	210 (53)	180 (45)
Nulliparous	999 (48)	170 (43)	188 (47)	179 (45)	206 (52)
Glucose tolerance
GDM	118 (6)	27 (7)	15 (4)	23 (6)	21 (5)
IGT	65 (3)	9 (2)	9 (2)	12 (3)	21 (5)
Failed GCT/normal OGTT	180 (9)	29 (7)	33 (8)	44 (11)	30 (8)
Normal	1,730 (83)	331 (84)	339 (86)	317 (80)	324 (82)
^***a***^Overall sample sizes for exposures are per Table 1; for all other characteristics, imputed data are shown (*n *= 2,093). Nonimputed data are available in Supplemental Material, Table S2. ^***b***^Spatiotemporal PM_2.5_ quartile ranges and sample sizes: Q1 (8.3–10.0 μg/m^3^; *n *= 396), Q2 (10.0–10.7 μg/m^3^; *n *= 396), Q3 (10.7–11.7 μg/m^3^; *n *= 396), Q4 (11.7–17.2 μg/m^3^; *n *= 396).					

In covariate-adjusted models, women in the highest (Q4) [vs. lowest (Q1)] quartile of second-trimester PM_2.5_ exposure had 1.90 times the odds (95% CI: 0.84, 4.31) of IGT versus normoglycemia when PM_2.5_ exposure was measured at the central monitoring station and 2.63 times the odds (95% CI: 1.15, 6.01) of IGT when PM_2.5_ was estimated by the spatiotemporal model. Odds of IGT versus normoglycemia for women in higher (Q2, Q3, Q4) versus the lowest (Q1) quartile of PM_2.5_ exposure were consistently positive when PM_2.5_ was measured at the central monitoring station and increased monotonically across quartiles when PM_2.5_ was estimated by the spatiotemporal model (Table 3). Results of unadjusted models were similar [e.g., odds of IGT for Q4 vs. Q1 were 1.82 (95% CI: 0.83, 3.99) for central-site PM_2.5_ and 2.40 (95% CI: 1.08, 5.31) for spatiotemporal PM_2.5_].

**Table 3:** Covariate-adjusted^*a*^ ORs (95% CIs) for failed GCT/normal OGTT, IGT, and GDM compared with normal glucose tolerance during pregnancy, by quartile (Q).

Exposure	Failed GCT/normal OGTT	IGT	GDM
Central-site PM_2.5 _(μg/m^3^)
Q1 (8.3–10.0)	1.0 (Reference)	1.0 (Reference)	1.0 (Reference)
Q2 (10.0–10.7)	1.15 (0.70, 1.90)	1.43 (0.62, 3.34)	0.91 (0.50, 1.65)
Q3 (10.7–11.7)	1.05 (0.64, 1.72)	1.44 (0.63, 3.29)	0.52 (0.27, 1.00)
Q4 (11.7–17.2)	1.31 (0.80, 2.13)	1.90 (0.84, 4.31)	0.69 (0.38, 1.27)
IQR (1.7)	1.15 (0.93, 1.41)	1.34 (0.98, 1.84)	0.81 (0.62, 1.08)
Spatiotemporal PM_2.5 _(μg/m^3^)
Q1 (8.5–10.8)	1.0 (Reference)	1.0 (Reference)	1.0 (Reference)
Q2 (10.8–11.8)	1.19 (0.69, 2.03)	1.14 (0.44, 2.95)	0.62 (0.30, 1.28)
Q3 (11.8–12.8)	1.71 (1.03, 2.84)	1.46 (0.60, 3.59)	0.93 (0.48, 1.78)
Q4 (12.8–15.9)	1.11 (0.64, 1.94)	2.63 (1.15, 6.01)	0.71 (0.35, 1.42)
IQR (2.0)	1.08 (0.84, 1.40)	1.64 (1.11, 2.42)	0.94 (0.67, 1.34)
Central-site black carbon (μg/m^3^)
Q1 (0.60–0.78)	1.0 (Reference)	1.0 (Reference)	1.0 (Reference)
Q2 (0.78–0.87)	0.98 (0.60, 1.62)	1.16 (0.54, 2.54)	0.75 (0.39, 1.45)
Q3 (0.87–0.94)	1.53 (0.82, 2.87)	2.41 (0.87, 6.69)	0.59 (0.25, 1.35)
Q4 (0.94–1.10)	1.18 (0.58, 2.40)	2.87 (0.93, 8.83)	0.60 (0.23, 1.53)
IQR (0.16)	1.11 (0.76, 1.63)	1.36 (0.74, 2.49)	0.69 (0.42, 1.13)
Spatiotemporal black carbon (μg/m^3^)
Q1 (0.14–0.55)	1.0 (Reference)	1.0 (Reference)	1.0 (Reference)
Q2 (0.55–0.70)	1.03 (0.67, 1.58)	1.39 (0.66, 2.96)	1.01 (0.54, 1.87)
Q3 (0.70–0.89)	1.03 (0.65, 1.63)	1.86 (0.87, 3.98)	1.12 (0.59, 2.09)
Q4 (0.89–1.69)	1.02 (0.62, 1.68)	1.50 (0.65, 3.50)	0.90 (0.45, 1.79)
IQR (0.34)	1.01 (0.79, 1.29)	1.09 (0.74, 1.62)	1.02 (0.73, 1.41)
Neighborhood traffic density^*b*^
Q1 (0–4,061)	1.0 (Reference)	1.0 (Reference)	1.0 (Reference)
Q2 (4,062–9,680)	1.51 (0.97, 2.36)	1.72 (0.79, 3.75)	1.18 (0.66, 2.11)
Q3 (9,680–19,371)	1.24 (0.78, 1.98)	1.04 (0.44, 2.48)	0.94 (0.51, 1.72)
Q4 (19,383–30,860)	1.38 (0.86, 2.21)	2.66 (1.24, 5.71)	0.74 (0.39, 1.42)
IQR (1,533)	1.12 (1.03, 1.23)	1.15 (1.00, 1.31)	1.02 (0.87, 1.18)
Home roadway proximity (m)
> 200	1.0 (Reference)	1.0 (Reference)	1.0 (Reference)
≤ 200	1.12 (0.69, 1.80)	1.83 (0.96, 3.51)	0.99 (0.52, 1.88)
^***a***^Adjusted for age, prepregnancy BMI, pregnancy weight gain, education, race/ethnicity, family history of diabetes, prior GDM, and season of last menstrual period. ^***b***^Vehicles/day × km road within 100 m.			

Women in the highest (vs. lowest) quartile of second-trimester black carbon exposure also had increased odds of IGT versus normoglycemia in covariate-adjusted models, but CIs included the null whether black carbon was measured at the central monitoring station (OR = 2.87; 95% CI: 0.93, 8.83) or estimated by the spatiotemporal model (OR = 1.50; 95% CI: 0.65, 3.50). Odds of IGT versus normoglycemia increased monotonically across quartiles when black carbon was measured at the central monitoring station and were consistently higher in Q2, Q3, and Q4 versus Q1 when black carbon was estimated by the spatiotemporal model (Table 3). In covariate-adjusted models, odds of IGT versus normoglycemia were also increased in women who lived in a neighborhood with the highest (vs. lowest) quartile of traffic density (OR = 2.66; 95% CI: 1.24, 5.71), although Q2, Q3, Q4 versus Q1 ORs did not increase monotonically, and, in fact, the Q3 versus Q1 comparison was close to 1. Women who lived ≤ 200 m (vs. > 200 m) from a major roadway also had increased odds of IGT vs normoglycemia (OR = 1.83; 95% CI: 0.96, 3.50) (Table 3).

**Figure 1:**
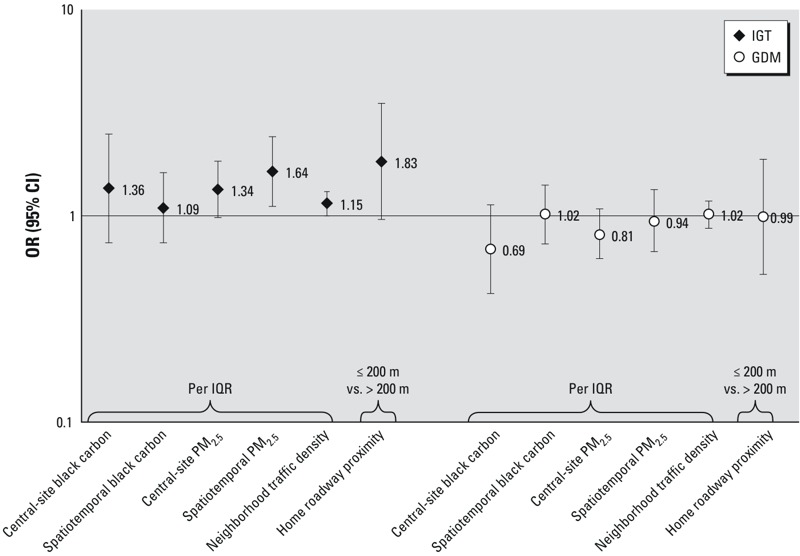
Associations of second-trimester exposure to PM_2.5_, second-trimester exposure to black carbon, neighborhood traffic density based on enrollment address, and home roadway proximity based on enrollment address, with risk for IGT and GDM during pregnancy. Data were from 2,093 Boston-area pregnant women in Project Viva. IQR, interquartile range. IQR = 0.16 μg/m^3^ for central-site black carbon, 0.34 μg/m^3^ for spatiotemporal black carbon, 1.7 μg/m^3^ for central-site PM_2.5_, 2.0 μg/m^3^ for spatiotemporal PM_2.5_, 1,533 vehicles/day × km for neighborhood traffic density.

We found no relationship between any exposure and GDM in either the unadjusted (data not shown) or covariate-adjusted models, with ORs for GDM generally < 1 (Table 3). When we represented PM_2.5_, black carbon, and traffic density exposures as continuous variables (per IQR), relationships with IGT were consistently positive and with GDM were consistently null (Figure 1).

In covariate-adjusted models, odds of a failed GCT/normal OGTT versus normoglycemia were not increased for women in the highest (vs. lowest) quartile of spatiotemporal PM_2.5_ (OR = 1.11; 95% CI: 0.64, 1.94) or other exposures (Table 3). When we included a variable for time trend or prepregnancy BMI squared to the final model, results were unchanged (data not shown). Inclusion of census tract median income slightly attenuated effect estimates for associations of IGT with spatiotemporal black carbon (OR for Q4 vs. Q1 = 1.24; 95% CI: 0.51, 3.05), spatiotemporal PM_2.5_ (2.4; 95% CI: 1.04, 5.53), and traffic density (2.41; 95% CI: 1.11, 5.25) but not for any other exposure–outcome relationships. When we restricted the analyses to women without prior GDM or to women with a measured rather than imputed outcome, results were also unchanged (data not shown). When we included both spatiotemporal PM_2.5_ and traffic density in the same adjusted model, odds of failed GCT/normal OGTT, IGT, or GDM versus normoglycemia per IQR increase in exposure were essentially unchanged. For example, an IQR increase in spatiotemporal PM_2.5_ exposure increased odds of IGT by 1.60 (95% CI: 1.08, 2.37) (vs. single-pollutant model OR = 1.64; 95% CI: 1.11, 2.42), and an IQR increase in traffic density increased odds of IGT by 1.17 (95% CI: 1.01, 1.35) (vs. single-pollutant model OR = 1.15; 95% CI: 1.0, 1.31).

## Discussion

Among pregnant women residing in the greater Boston area, second-trimester PM_2.5_ exposure was positively associated with IGT, but not frank GDM. Second-trimester black carbon exposure, and traffic density and roadway proximity based on enrollment address also appeared to be associated with IGT.

Our results are consistent with previous studies suggesting glycemic effects of air pollution. Long-term exposure to PM_2.5_ and PM_10_ (PM with diameter < 10 μm) and self-reported type 2 diabetes mellitus has been studied in several adult cohorts (Andersen et al. 2012; Brook et al. 2008; Coogan et al. 2012; Krämer et al. 2010; Pearson et al. 2010; Puett et al. 2011), of which all but one (Puett et al. 2011) reported positive associations between diabetes and particulate matter exposures. Studies of short-term PM_2.5_ and PM_10_ exposure in adults have also demonstrated an association between exposure during the days before a blood draw and biochemical measures of insulin resistance (Brook et al. 2013; Kim and Hong 2012).

Two prior studies have considered air pollution exposure and glycemia in pregnancy, and results were conflicting. A cohort study of > 7,000 pregnant women in the Netherlands (van den Hooven et al. 2009) found no association between traffic density and GDM. In contrast, a study of birth registry data for > 81,000 births in Sweden (Malmqvist et al. 2013) reported monotonic dose–response associations between NO_x_ and GDM, and positive associations with traffic density.

In rodent models, PM_2.5_ exposure resulted in greater oxidative stress and adipose tissue inflammation [reviewed by Anderson et al. (2012); Franchini et al. (2012)]. Adipose inflammatory changes included increased proinflammatory to antiinflammatory macrophage ratio and insulin signaling abnormalities, which could lead to insulin resistance (Sun et al. 2009; Xu et al. 2011). Obesity-induced insulin resistance is thought to similarly occur as a direct result of adipose inflammation (Ye 2013).

It is uncertain whether the traffic components of PM (e.g., black carbon) are responsible for these associations. Traffic and nontraffic PM components have been associated with increases in systemic inflammatory markers and with adverse vascular responses in adults with diabetes (Gold 2008; O’Neill et al. 2005). In the present study, an IQR increase in PM_2.5_ exposure (estimated in spatiotemporal models) was more strongly associated with IGT than an IQR increase in traffic density when both variables were included in the same adjusted model. Although this difference could reflect different degrees of measurement error in the exposure variables, it may also suggest a greater impact of nontraffic PM versus traffic-related PM on IGT. Traffic density captures spatial variability from locally generated air pollution but lacks temporal resolution; therefore, it may have been less strongly related to IGT because of inadequate capture of regional particle movement occurring specifically during a woman’s second trimester of pregnancy.

Although there was an association of air pollution exposure with IGT, contrary to our hypothesis, we did not demonstrate an association with frank gestational diabetes. In fact, ORs for GDM were generally < 1, although CIs all included the null. This result is consistent with several studies that have reported gestational weight gain, another modifiable exposure during gestation, to be associated with IGT but not GDM (Herring et al. 2009; Saldana et al. 2006; Tovar et al. 2009). Mothers predisposed to eventually develop GDM may enter pregnancy with an array of preexisting risk factors such as greater pregravid weight and family history of diabetes (Solomon et al. 1997) and may develop GDM regardless of additional behavioral or environmental risk factors during pregnancy. Thus, more severe degrees of hyperglycemia may be less sensitive to short-term exposures. Another possible explanation for the differential results for IGT versus GDM is that individuals with undiagnosed, preexisting diabetes may have been included in the GDM group, thus limiting our ability to demonstrate an association with pregnancy-specific exposures. In any case, it will be necessary to replicate this finding in future work, because prior studies of air pollution and glucose tolerance in pregnancy (Malmqvist et al. 2013; van den Hooven et al. 2009) did not include a separate IGT designation.

Even mild degrees of abnormal glycemia in pregnancy (i.e., IGT) have been associated with adverse perinatal clinical outcomes (Hapo Study Cooperative Research Group et al. 2008; Sermer et al. 1995) and future obesity and insulin resistance in both mother and offspring (Hillier et al. 2007). To put our findings into perspective, the extent to which second-trimester spatiotemporal PM_2.5_ exposure increased odds of IGT in the present study (OR = 2.63; 95% CI: 1.15, 6.01, for highest vs. lowest quartile of exposure) is of the same order of magnitude as that of other well-known risk factors for IGT. For example, in our cohort, the OR of IGT was 2.54 (95% CI: 1.25, 5.15) for women in the highest versus lowest quartile of gestational weight gain, and was 1.89 (95% CI: 1.04, 3.44) for those with pregravid overweight versus normal weight (Herring et al. 2009).

Potential exposure misclassification is a limitation of the present study. The PM_2.5_ spatiotemporal model we used had a high mean out-of-sample *R*^2^, and use of satellite aerosol optical depth data was a strength, but air pollution estimates (for both PM_2.5_ and black carbon models) were based on residential address, and we did not have information on work location or time spent at home which could have improved the accuracy of exposure estimates (Nethery et al. 2008). Also, the PM_2.5_ spatiotemporal model estimated 10 × 10 km exposures, which could limit local contrast. However, the 2,093 women lived in 110 different 10 × 10 km cells throughout eastern Massachusetts, so there was still broad exposure variability. Also, the number of women with PM_2.5_ spatiotemporal estimates based on satellite data, which was not available before March 2000, was limited. Compared with those with available estimates, women missing spatiotemporal PM_2.5_ estimates had differences in their exposure profile, likely as a result of changing exposures over time. However, exposure differences would not be expected to bias results, as these women did not differ in terms of sociodemographic characteristics or proportion of IGT or GDM. Because the women were pregnant from 1999 through 2002, our use of 2009 traffic density in the black carbon spatiotemporal model may have increased exposure misclassification. Another limitation is use of self-reported prepregnancy weight, which may be underestimated, but a prior validation study of 170 Project Viva participants with measured prepregnancy weight suggested that ranking of individuals is preserved, and weight reporting did not differ by BMI or race/ethnicity (Oken et al. 2007). Also, generalizability may be limited because our cohort was older and mostly white, although the proportions of racial/ethnic minorities in Project Viva were higher than in Massachusetts as a whole, according to the 2000 census (U.S. Census Bureau 2000b). Strengths of our study include use of a large, prospective cohort with several measures of air pollution exposure and inclusion of multiple potential confounding variables. However, we did not account for every factor that might be related to pollution exposure and GDM risk, such as physical activity.

## Conclusions

In summary, second-trimester PM_2.5_ exposure was associated with impaired glucose tolerance, but not GDM, among pregnant women. Our results lend support to the emerging body of evidence that air pollution exposure is associated with abnormal glycemia.

## Supplemental Material

(164 KB) PDF
