# Supplementary material for: Air Pollution Exposure and Abnormal Glucose Tolerance during Pregnancy: The Project Viva Cohort
Source: Environ Health Perspect. 2014 Feb 7;122(4):378–83. doi: 10.1289/ehp.1307065 (PMC3984217; doi:10.1289/ehp.1307065)
Supplement: (164 KB) PDF [file ehp.1307065.s001.pdf]

Environmental Health Perspectives doi:10.1289/ehp.1307065

Supplemental Material

Air Pollution Exposure and Abnormal Glucose Tolerance during Pregnancy: The Project Viva Cohort

Abby F. Fleisch, Diane R. Gold, Sheryl L. Rifas-Shiman, Petros Koutrakis, Joel D. Schwartz, Itai Kloog, Steven Melly, Brent A. Coull, Antonella Zanobetti, Matthew W. Gillman, and Emily Oken

Table of Contents **Page**

Table S1 2

Table S2 3

Table S1. Correlations between exposures (Spearman r).

**Exposure**	**$PM_{2.5}$ central-site**	**$PM_{2.5}$ spatio-temporal**	**Black carbon central-site**	**Black carbon spatio-temporal**	**Traffic density**	**Roadway proximity**
**$PM_{2.5}$**						
Central-site	1					
Spatiotemporal	0.79	1				
**Black carbon**						
Central-site	0.24	0.19	1			
Spatiotemporal	0.08	0.39	0.36	1		
**Traffic density**	-0.002	0.16	-0.03	0.50	1	
**Roadway proximity**	0.01	-0.19	0.01	-0.34	-0.38	1

Table S2. Characteristics of participants with[a] and without imputed data and with and without spatiotemporal $PM_{2.5}$ exposure data.

	Mean ± SD or % with imputation	N without imputation	Mean ± SD or % without imputation	N with $PM_{2.5}$ data	Mean ± SD or % with $PM_{2.5}$ data	N without $PM_{2.5}$ data	Mean ± SD or % without $PM_{2.5}$ data	p-value
**Maternal age at enrollment (years)**	31.8 ± 5.2	2,093	31.8 ± 5.2	1,584	31.9 ± 5.2	509	31.4 ± 5.4	0.07
**Prepregnancy BMI (kg/m^2)**	24.9 ± 5.6	2,078	24.9 ± 5.5	1,574	24.8 ± 5.4	504	25.1 ± 6.0	0.34
**Pregnancy weight gain to OGTT (kg)**	10.2 ± 4.4	2,023	10.2 ± 4.4	1,556	10.3 ± 4.4	467	10.2 ± 4.3	0.66
**Central-site $PM_{2.5}$ (μg/m^3)**	N/A	1,943	10.9 ± 1.4	1,466	11.2 ± 1.4	477	10.2 ± 1.2	<0.001
**Spatiotemporal $PM_{2.5}$ (μg/m^3)**	N/A	1,584	11.9 ± 1.4	1,584	11.9 ± 1.4	N/A	N/A	
**Central-site black carbon (μg/m^3)**	N/A	1,943	0.9 ± 0.1	1,466	0.8 ± 0.1	477	0.9 ± 0.1	<0.001
**Spatiotemporal black carbon (μg/m^3)**	N/A	2,069	0.7 ± 0.2	1,571	0.7 ± 0.2	498	0.8 ± 0.3	<0.001
**Traffic density [(vehicles/day) x km]**	N/A	2,081	1,621 ± 2,234	1,574	1,625 ± 2,198	507	1,608 ± 2,344	0.89
**Roadway proximity (≤ 200 m)**	N/A	2,092	13%	1,583	13%	509	14%	0.92
**College graduate**	65%	2,069	65%	1,570	66%	499	62%	0.16
**Race/ethnicity**		2,069		1,570		499		
White	67%		67%		67%		66%	0.11
Black	17%		16%		16%		19%	
Asian	6%		6%		6%		6%	
Hispanic	7%		7%		8%		5%	
Other	4%		4%		4%		4%	
**Family history of diabetes**	8%	1,719	7%	1,325	8%	394	7%	0.61
**Prior history of gestational diabetes**		2,063		1,565		498		0.27
Yes	2%		2%		2%		2%	
No	50%		50%		51%		46%	
Nulliparous	48%		48%		48%		51%	
**Glucose tolerance**		2,050		1,574		476		0.86
GDM	6%		6%		55%		6%	
IGT	3%		3%		3%		3%	
Failed GCT/normal OGTT	9%		9%		9%		9%	
Normal	83%		83%		83%		82%	

[a]Only covariates and outcomes were imputed. N = 2,093 for all imputed data.
